# Transcriptome-Wide Identification and Characterization of Circular RNAs in Leaves of Chinese Cabbage (*Brassica rapa* L. ssp. *pekinensis*) in Response to Calcium Deficiency-Induced Tip-burn

**DOI:** 10.1038/s41598-019-51190-0

**Published:** 2019-10-10

**Authors:** Wuhong Wang, Jinglei Wang, Qingzhen Wei, Biyuan Li, Xinmin Zhong, Tianhua Hu, Haijiao Hu, Chonglai Bao

**Affiliations:** Institute of Vegetable Research, Zhejiang Academy of Agricultural Sciences, Hangzhou, 310021 China

**Keywords:** Non-coding RNAs, Abiotic

## Abstract

Circular RNA (circRNA) is a newly discovered non-coding RNA, which play significant roles in the function and transcriptional regulation of microRNA. To date, in Chinese cabbage, the functional characteristic of circRNAs in response to calcium deficiency-induced tip-burn have not been reported. In this study, 730 circRNAs were isolated from Chinese cabbage leaves, of which 23 and 22 were differentially expressed in different calcium deficiency stages compared with the control. Forty-six host genes of the differentially expressed circRNAs were identified, and one circRNA was found to act as miRNAs sponges. Based on the functional analysis of host genes and target mRNAs of the corresponding miRNAs, the identified circRNAs might participated in response to stimulus, electron carrier activity, ATPase activity, cell wall metabolism, transcription factors and plant hormone signal transduction. *ABF2*, a positive regulator of the abiotic stress response in the abscisic acid (ABA) pathway, may play a role in calcium deficiency tolerance through a circRNA regulatory pathway. Correspondingly, the concentration of ABA is also increased during the Ca^2+^ deficiency stress. Our results suggest that circRNAs participate in a broad range of biological processes and physiological functions in the response to calcium deficiency-induced tip-burn and provide a basis for further studies of the biological roles that circRNAs play in the plant stress response.

## Introduction

Chinese cabbage (*Brassica rapa* L. ssp. *pekinensis*), a member of the Brassiceae tribe in the Brassicaceae family, has a long cultivation history and is an economically important vegetable grown worldwide. Tip-burn, which is generally considered a calcium-associated physiological disorder^1,2^, occurs frequently and has become one of the major diseases posing a serious threat to Chinese cabbage yield and quality in recent years^3^. Characteristic symptoms of tip-burn initially necrosis occurs at tip and margin of leaves, then, as the disease progresses, the whole leaf became brown and shrunk. Moreover, tip-burn is always accompanied by pathogen infections, which further reduce production^4^.

Although the mechanisms of tip-burn are both complex and controversial, the inability of a plant to supply rapidly developing leaves with sufficient calcium (Ca^2+^) is generally accepted as a contributing factor^5^. In plant cells, Ca^2+^ is primarily stored in mesophyll cell vacuoles; however, the intracellular Ca^2+^ shows significant levels of modulation in responding to various stress signals^6–8^. Cytosolic Ca^2+^ homeostasis is controlled by Ca^2+^ storage and transport system, including influx and efflux^9^. The best-characterized Ca^2+^ transporters are ACA (P_2B_-type Ca^2+^-ATPase pump), CAX (Ca^2+^/H^+^ antiporter) and ECA (P_2A_-type Ca^2+^-ATPase pump) proteins^10–12^. Three groups of ACAs are localized in the plasma membrane, endoplasmic reticulum (ER), and tonoplast^13–15^. In *A. thaliana*, only *ACA4* and *ACA11*, which are expressed in the leaf tissue and localized on the tonoplast, are confirmed as important for removing excess cytoplasmic Ca^2+^ to vacuoles in experiments. With a double-knockout mutation of *ACA4* and *ACA11*, scattered lesions appeared around the leaves, particularly at the leaf margin^16^. Of the 11 CAX antiporters (CAX 1–11) identified in *Arabidopsis*, only CAX 1–4 are functionally characterized to have an activity to exchange vacuolar Ca^2+^/H^+^^17–20^. CAX2 and CAX4 can transport a range of cations but do not act as main physiological role in Ca^2+^ homoeostasis^20–22^. The *cax1/cax3* double mutation exhibits necrosis of the leaf tips and shoot apex, and CAX1 and CAX3 antiporters can exchange one cytoplasmic Ca^2+^ with two vacuolar protons^23^. The ECA pumps (ECA1, ECA3 and ECA4) in endomembranes are essential for the balance of Ca^2+^/Mn^2+^ between the cytoplasm and the ER, which serves as a central coordinator of plant development and adaptation to stress^24–26^. Additionally, the expression of Ca^2+^-metabolism-related genes including *CAX1*, *ACA4*, and *ACA11* responds differently to abiotic stresses in different tip-burn-resistant *Brassica oleracea*^27,28^. These results indicated that ACA, CAX, and ECA transporters participate in the process of Ca^2+^ deficiency-induced tip-burn.

Previous studies show that non-coding RNA genes also participate in responding to disease and stress in plants, including dehydration responsive microRNAs (miRNAs)^29^ and cadmium stress responsive lncRNAs^30^. Circular RNAs (circRNAs) is a newly discovered endogenous non-coding RNAs that lack 5′ or 3′ ends similar to miRNAs. Evidence is currently emerging that circRNAs may regulate the function of microRNA (miRNA) and its roles in transcriptional control^31^. Besides, circRNAs are reported always show tissues- or developmental-stage-specific expression patterns^32^. Although the functions of circRNAs remain not very clear, their evolutionary conservation, high expression abundance and origin from important gene loci imply that they have vital functions in plants^33^. Recently, several studies propose that the circRNAs play important regulatory roles in responding to environmental stress in rice^34^, wheat^35^ and tomato^36^. To our knowledge, reports about the role of circRNAs in the stress response of Brassica vegetables continues to be lacking.

To explore the prevalence of circRNAs in Chinese cabbage and their potential roles in response to Ca^2+^ deficiency-induced tip-burn, we performed genome-wide analysis of circRNAs in Chinese cabbage leaves using Illumina next sequencing technology under normal and Ca^2+^ deficiency stress. Moreover, the differentially expressed circRNAs were identified and their potential function were further analysed and discussed in this study.

## Results

### Antioxidant enzymes and ABA concentration analysis during calcium deficiency-induced tip-burn

**Figure 1:**
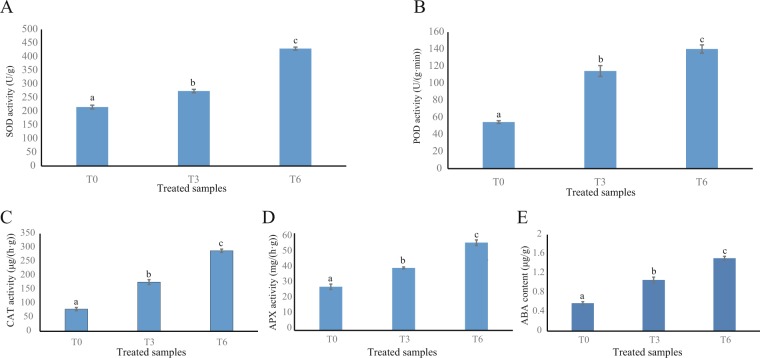
Changes in the enzyme activities of SOD, POD, CAT, APX and ABA concentrations in *B. rapa* leaves during calcium deficiency-induced tip-burn. Values represent mean ± SE. T0 present leaf tissues without treatment, T3 and T6 presents 3 and 6 d after treatment, respectively. Lowercase letters ‘a, b, c’ show significant differences (p < 0.05).

In response to abiotic stresses, plants always accumulate various antioxidant enzymes including SOD, CAT, POD and APX to quench free radicals, which including O_2_^−^ and H_2_O_2_^37^. The activities of all 4 enzymes were increased within 6 days under Ca^2+^ deficiency stress (Fig. 1A–D). ABA is known to play a pivotal role in plants against stress. In current study, the concentration of ABA is also increased during the Ca^2+^ deficiency stress (Fig. 1E), which is consistent with the physiological indexes of increased activities of antioxidant enzymes.

### Identification of circRNAs in chinese cabbage

In total, 65.66 Gb of clean data was generated from the 9 samples, and the Q30 of the clean reads was more than 91.30% in each library, indicating that high-quality clean reads were obtained in this study. The clean data was mapped to *B. rapa* reference genome sequence, resulting in the ratios of uniquely mapped reads ranging from 96.16 to 98.56% (Table 1). These results revealed that the sequence data were qualified for further circRNA analysis. The circRNA-seq data files have been deposited in the NCBI Sequence Read Archive (SRA) database (BioProject PRJNA557368).

**Table 1:** Detailed information of sequenced data for each sample.

Samples	Read Numbers	Base Numbers	GC Content	% ≥ Q30	Mapped Reads
T0-1	27,983,426	8,361,356,794	43.42%	91.66%	99.33%
T0-2	24,868,398	7,435,559,120	43.00%	91.67%	99.81%
T0-3	24,732,169	7,394,753,602	42.79%	91.67%	99.78%
T3-1	21,294,563	6,362,688,772	43.35%	91.60%	99.76%
T3-2	22,538,462	6,740,229,522	43.39%	91.72%	99.71%
T3-3	21,680,976	6,486,662,744	42.61%	91.57%	99.77%
T6-1	24,133,763	7,221,308,600	43.05%	91.49%	99.81%
T6-2	26,491,888	7,929,780,532	43.25%	91.38%	99.77%
T6-3	25,863,064	7,729,178,524	44.21%	91.30%	99.70%

**Figure 2:**
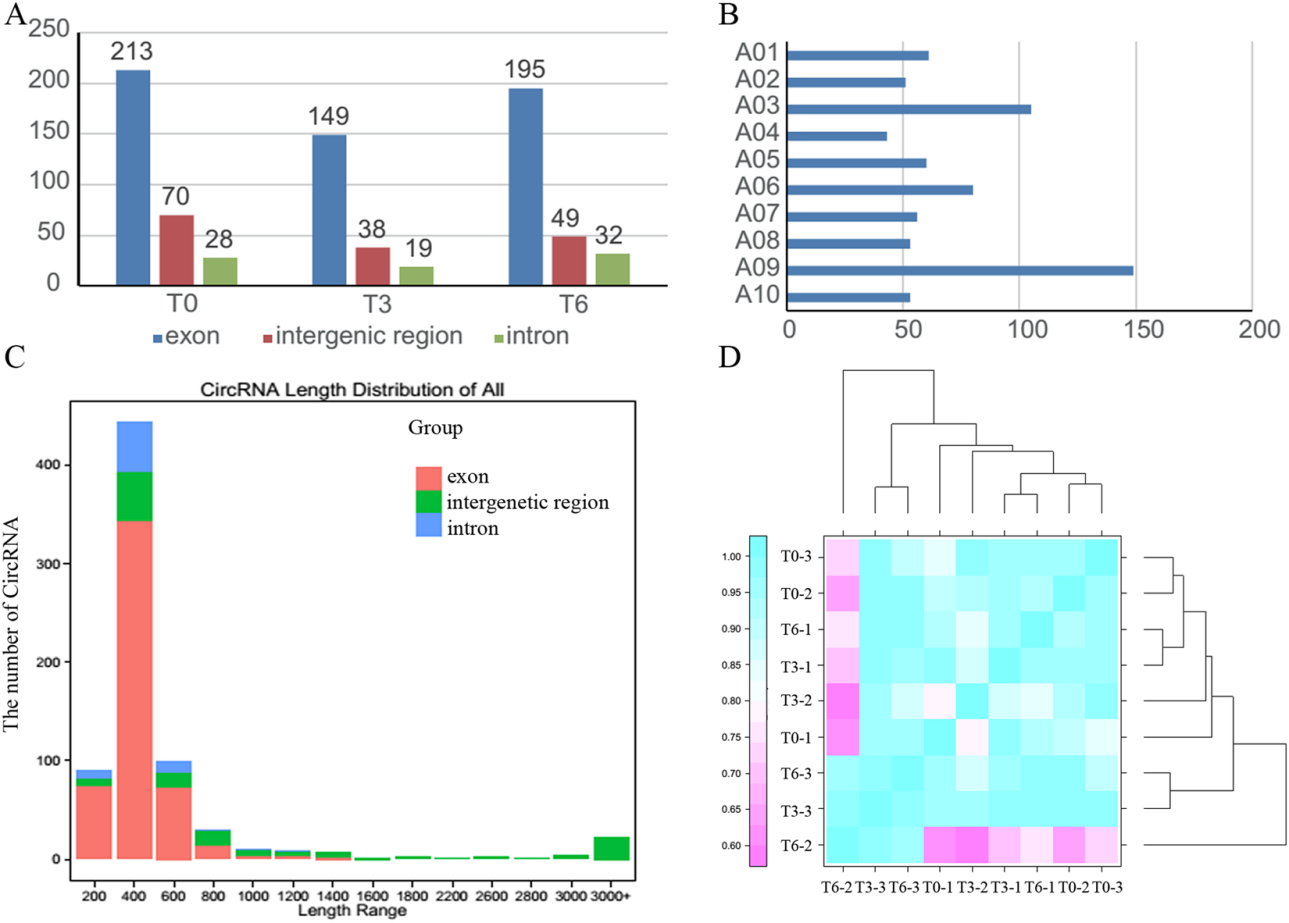
Characterization of Chinese cabbage circRNAs. (**A**) Derivation distribution of the identified circRNAs in the three treatments. (**B**) The chromosome distribution of the circRNAs. (**C**) Length distribution of circRNAs in Chinese cabbage. (**D**) The correlation coefficients for biological repeats in the three treatments.

Based on the sequence reads, the number of candidate circRNAs identified in the 3 treatments ranged from 206 to 311. circRNAs are classified into intergenic, exon and intron groups according to their origin. Among the total 730 circRNAs identified in Chinese cabbage, most (68.49, 72.33 and 70.65% in the T0, T3 and T6 treatments, respectively) were exonic circRNAs, 22.51, 18.45 and 17.75% were intergenic circRNAs in the three treatments, and the remaining were intronic circRNAs (Fig. 2A). Regarding chromosome distribution, 19 of the 730 circRNAs were produced from Scaffolds, and chromosome A09 have the most circRNAs, followed by chromosome A03 (Fig. 2B and Supplementary Table S1). It is noteworthy that the length distribution of these circRNAs ranged from 109 to 23,033 bp. The most abundant lengths were in the range from 200 to 600 bp, and the long circRNAs with more than 1600 bp were generated from intergenic regions (Fig. 2C). Correlation coefficients among the biological repeats each exceeded 0.76, indicating the data were reliable for further analysis (Fig. 2D).

### The expression patterns of circRNAs in chinese cabbage during calcium deficiency-induced tip-burn

**Figure 3:**
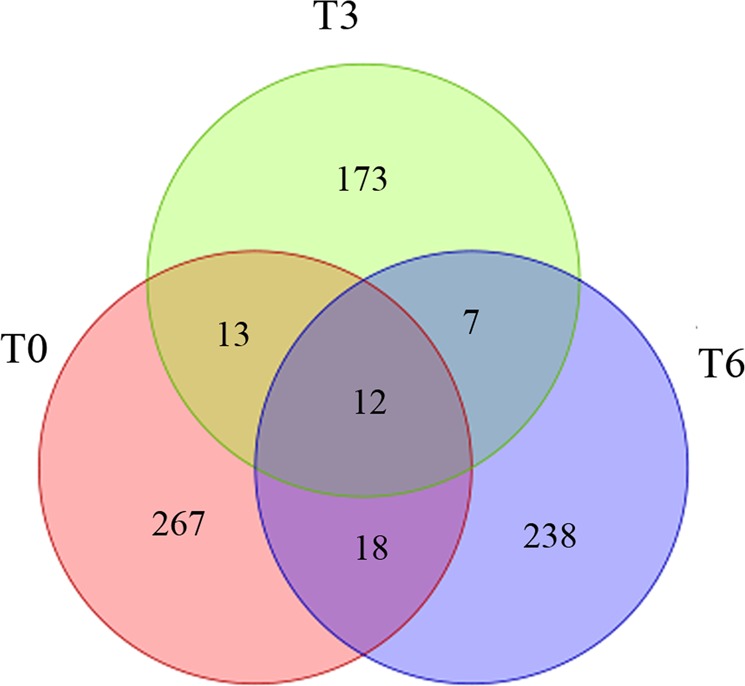
Venn diagram of identified circRNAs of the three stages in Chinese cabbage during calcium deficiency-induced tip-burn.

To investigate the circRNAs biological function during calcium deficiency-induced tip-burn, we compared the expression levels of circRNAs among the three stages. Remarkably, most circRNAs seem to be specifically expressed in one stage. Specifically expressed were 267, 173 and 238 circRNAs in the stages T0, T3 and T6, respectively. However, for stages T0 and T3, only 13 circRNAs were shared, and for stages T3 and T6, only 7 circRNAs were shared. The three stages shared 12 circRNAs (Fig. 3). Additionally, 23 circRNAs were significantly different between stages T0 and T3, with 13 circRNAs up regulated and 10 circRNAs down regulated, and 22 circRNAs were significantly differentially expressed between stages T0 and T6, with 11 circRNAs up regulated and 11 circRNAs down regulated (Supplementary Table S2). The results indicated that circRNAs have specific roles in the response of calcium deficiency-induced tip-burn.

### Putative biological function of circRNAs according to their host genes

The circRNAs play significant roles in transcriptional control by *cis-*regulation of their host genes^38^. The host genes of differentially expressed circRNAs were predicted and annotated. A total of 46 host genes were identified when the host genes of differentially expressed circRNAs of the three stages were combined.

**Figure 4:**
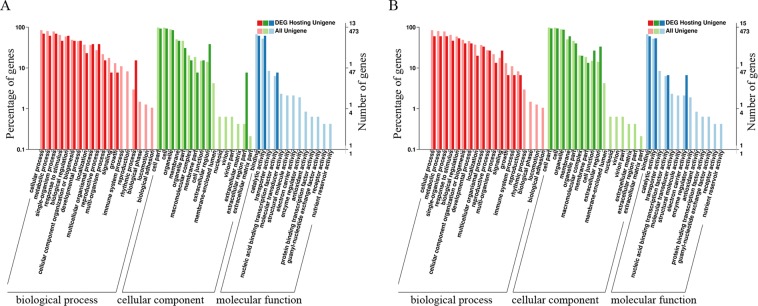
The Gene Ontology (GO) classification of host gene annotations of differentially expressed circRNAs. GO terms are summarized in three categories, namely biological process, cellular component and molecular function. (**A**) The GO classification of host genes of differentially expressed circRNAs between the T0 and T3 stages. (**B**) The GO classification of host genes of differentially expressed circRNAs between the T0 and T6 stages.

In order to understand the biological function of the different expressed circRNAs, GO analysis was performed on their host genes. A total of 9, 13, and 10 host genes of differentially expressed circRNAs between the T0 and T3 stages were classified according at the first level of classification into biological processes, molecular functions and cellular components, respectively. Additionally, 10, 15, and 10 host genes of differentially expressed circRNAs between the T0 and T6 stages were classified into three categories. The two GO classifications both identified cellular process, metabolic process, response to stimulus, signalling, organelle, membrane, extracellular region, binding, catalytic activity and some other categories. Notably, rhythmic process and extracellular region part were only found in the differentially expressed circRNA GO classification of T0 and T3 stages, and electron carrier activity was only found in the classification of T0 and T6 stages (Fig. 4 and Supplementary Table S3).

The host genes were further annotated by using the KEGG database. Three pathways of plant hormone signal transduction, peroxisome and carbon metabolism were identified in both host gene sets of differentially expressed circRNAs between the T0 and T3 stages and between the T0 and T6 stages. Notably, the different plant hormone signal pathways were clustered between the two differentially expressed gene sets. In the T0 and T3 stages, abscisic acid (ABA) responsive element binding factor (K14432) was involved. However, brassinosteroid-resistant protein (K14503) and auxin-responsive protein (K14484) were involved in the T0 and T6 stages. Moreover, the host genes of differentially expressed circRNAs were also involved in biosynthesis of amino acids in T0 and T3 stages and in non-homologous end-joining and RNA transport in T0 and T6 stages (Supplementary Table S4).

### Putative functions of circRNAs acting as putative miRNA sponges

CircRNAs play import roles in regulating the expression of functional genes by acting as competing miRNA sponges, which prevent the miRNAs from regulating their target mRNAs^33^. We found that a total of 25 circRNAs have predicted miRNA-binding sites, and 26 corresponding miRNAs were identified (Supplementary Table S5). Except for circRNAs Scaffold000096:707852|715672 and A09:20102134|20102717, which had 7 and 3 miRNA-binding sites, respectively, the others had only one miRNA-binding site. Moreover, 1229 mRNAs were identified as the targets of the 26 miRNAs. The number of the target mRNAs of miRNAs ranged from 6 to 178 (Supplementary Table S6).

Of the 25 circRNAs, only one (A03:5084249|5089986) was differentially expressed in stages T0 and T3, T0 and T6. Bra-miR5716, which was the corresponding miRNA of A03:5084249|5089986, had 15 target mRNAs in Chinese cabbage. The functions of the target mRNAs included ATPase activity, oxidation-reduction, electron carrier activity, transmembrane transport and Myb-type HTH DNA-binding (Supplementary Table S7).

### qRT-PCR validation of circRNA-seq results

**Figure 5:**
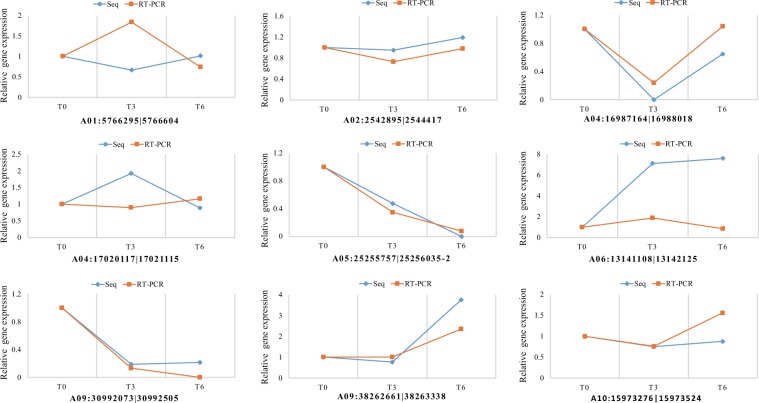
Quantitative real-time PCR validations of circRNAs expression levels from circRNA-seq analysis.

To validate the accuracy and reliability of circRNA-seq transcriptome results, nine highly expressed circRNAs used in qRT-PCR. The primers of 18 S rRNA and selected circRNAs were attached in Supplementary Table S8. Except for three circRNAs A01:5766295|5766604, A04:17020117|17021115 and A06:13141108|13142125, other six showed similar expression patterns detected by the two methods, although change folds varied in different experiments (Fig. 5).

## Discussion

In the last few years, a large number of distinct circRNAs have been discovered in various animals and model plants using “Next-generation” sequencing technology and bioinformatics analysis. Comparing previous reports on other higher plants, 2354, 5372, 2098 and 854 circRNAs were identified from rice^34^, soybean^39^, potato^40^, and tomato^36^, respectively. Therefore, the 730 identified circRNAs are fewer than those in other crop plants in many previous reports, but the number is similar to that in tomato. Among the identified circRNAs in Chinese cabbage, approximately 19.57% were intergenic circRNAs and 70.49% were exonic circRNAs, which are proportions similar to those in *Arabidopsis*, rice, and tomato. However, in wheat, potato and kiwifruit, most of the circRNAs were intergenic circRNAs, suggesting that circRNA origin is species-specific^41^.

With the three samples at 0, 3 and 6 d after Ca^2+^ deficiency treatment, we could identify changes in the circRNAs expression at different stages and trace the underlying responding circRNAs to calcium deficiency-induced tip-burn in Chinese cabbage. Analysis the host genes of circRNAs is one of the primary methods to provide valuable hints regarding the functions of circRNAs, considering that the function of only a few circRNAs has been clarified^42^. Therefore, we conducted GO and KEGG analysis on host genes of the different expressed circRNAs in this study. The biological interpretation of these host genes included cellular process, metabolic process, response to stimulus, electron carrier activity, plant hormone signal transduction, and ATPase activity, among others. These results suggested that circRNAs were participated in a broad range of biological processes and physiological functions in the calcium deficiency-induced tip-burn response. Although some host genes and their annotation were shared between the two differentially expressed circRNA sets of T0 and T3 and T0 and T6 stages, several functions were specifically annotated in each set, which indicated that the response of circRNAs to calcium deficiency-induced tip-burn was dynamic in Chinese cabbage.

Cell wall metabolism is an important facet of basal stress and disease responses^43^, and calcium deficiency can affect the components of cell wall structure^44^. In this work, cell wall modification (GO:0042545) and plant-type cell wall modification (GO:0009827) were obtained. Simultaneously, glucan metabolic process (GO:0044042) and cellular glucan metabolic process (GO:0006073) were also obtained. Previous study shows that cell wall glucan content changes and glucan synthetase activity is affected in calcium deficiency-treated Chinese cabbage leaf^45^. Hence, we speculate that circRNAs are involved in the response to tip-burn through cell wall metabolism.

Many phytohormones, including abscisic acid, brassinosteroids, and salicylic acid, play vital roles in the ability of plants to respond to abiotic and biotic stress^46^. Numerous studies show the critical roles of ABA in acting toward various stresses such as heavy metals, drought, salinity, low temperature, and radiation stress^47^. In this study, ABA also accumulated during the Ca^2+^ deficiency stress, which suggested ABA have a regulatory role in response to Ca^2+^ deficiency-induced tip-burn in *B. rapa*. *ABF2* (Bra040260), which is Ca^2+^-regulated^48,49^, was a host gene of differentially expressed circRNA between T0 and T3 stages. *ABF2* is also a positive regulator of the abiotic stress response and ABA-dependent signalling transduction pathway in rice^50^ and rapeseed^51^. Brassinosteroids, which can induce stomatal closure, is similar to the action of ABA^52^. Exogenous application of brassinosteroids in agriculture can protect crops from various stress injuries to improve growth and yield^53,54^. Auxins play a role in drought tolerance through a miRNA regulatory pathway^54,55^, and a faint auxin signal can influence the synthesis and reaction of other phytohormones because of the cross-talk among the plant hormone pathways^56^. The host genes of differentially expressed circRNAs between T0 and T6 stages were involved in brassinosteroid and auxin pathways. Previous studies demonstrate that circRNAs are involved in dehydration and chilling response processes through plant hormone signal transduction involving auxins, brassinosteroids, jasmonic acid and ABA in rice and tomato^35,36^. Hence, circRNAs might function in responding to various abiotic stresses through hormone signal pathways in many plants.

Previous studies reveal that circRNAs play pivotal roles in miRNA function and transcription regulation by acting as competing endogenous RNAs^32,41^. Here, we found that 25 circRNAs contained miRNA-binding sites; however, only one was differentially expressed. Similar to the annotation in the parent genes of circRNAs, ATPase and transmembrane transport were obtained. Moreover, two transcription factors were identified, including MYB (Bra035457) and RLK (Bra036977). Previous studies demonstrate that MYB and RLK proteins play vital roles in plant responses to various kinds of abiotic stresses^57,58^. Our results further implied that circRNAs might participate in the calcium deficiency stress response via miRNA pathways.

Moreover, response to stimulus, electron carrier activity and ATPase activity were also obtained. ATPases not only involved in uptake of Ca^2+^ ions but also play a role in transport of those ions in root cells^59^. Additionally, except for the function of some predicted host or target genes of the differentially expressed circRNAs associated with the response to calcium deficiency-induced tip-burn, involvement of several was not experimentally demonstrated until this study, with functions such as biosynthesis of amino acids, non-homologous end-joining, and RNA transport, among others. For their possible roles in the response to calcium deficiency, the aspects of circRNA regulation also deserve attention.

## Material and Methods

### Plant materials and Ca^2+^ deficiency treatments

The seeds of Chinese cabbage were germinated at 25 °C in an incubator. The plant seedlings were transferred to a liquid culture medium that consisted of improved Hoagland’s medium at pH ∼6.0^60^. The whole plants were cultured in a light incubator under a 16:8 h (light: dark) photoperiod at 28 °C. At the four-leaf stage, the plants were moved to purified water and cultivated for 4 days. Then, the plants were transferred to Hoagland’s nutrient solution without Ca^2+^ according to Wang *et al*.^60^, which moved Ca(NO_3_)_2_ and changed the concentration of NH_4_NO_3_, KNO_3_, NH_4_H_2_PO_4_ to 2, 10 and 2 mmol/L, respectively. The improved Hoagland’s medium contained Ca(NO_3_)_2_·4H_2_O 945 mg/L, NH_4_H_2_PO_4_ 115 mg/L, KNO_3_ 506 mg/L, MgSO_4_·7H_2_O 493 mg/L, KH_2_PO_4_ 136 mg/L, NH_4_NO_3_ 80 mg/L, KI 0.83 mg/L, H_3_BO_3_ 6.3 mg/L, MnSO_4_·4H_2_O 22.3 mg/L, ZnSO_4_·7H_2_O 8.6 mg/L, Na_2_MoO_4_·2H_2_0 0.25 mg/L, CuSO_4_·5H_2_0 0.025 mg/L, COCl_2_·6H_2_O 0.025 mg/L, FeSO_4_·7H_2_O 27.3 mg/L and Na_2_-EDTA 37.3 mg/L. Hoagland’s nutrient solution without Ca^2+^ was consisted of NH_4_H_2_PO_4_ 230 mg/L, KNO_3_ 1010 mg/L, MgSO_4_·7H_2_O 493 mg/L, KH_2_PO_4_ 136 mg/L, NH_4_NO_3_ 160 mg/L, KI 0.83 mg/L, H_3_BO_3_ 6.3 mg/L, MnSO_4_·4H_2_O 22.3 mg/L, ZnSO_4_·7H_2_O 8.6 mg/L, Na_2_MoO_4_·2H_2_0 0.25 mg/L, CuSO_4_·5H_2_0 0.025 mg/L, COCl_2_·6H_2_O 0.025 mg/L, FeSO_4_·7H_2_O 27.3 mg/L and Na_2_-EDTA 37.3 mg/L. The comparation of elements concentration between the improved Hoagland’s medium and Hoagland’s nutrient solution without Ca^2+^ clearly displayed in Table 2.

**Table 2:** The elements concentration of the improved Hoagland’s medium and Hoagland’s nutrient solution without Ca^2+^.

Element	Ca	N	K	P	Mg	B	Mn	Zn	Mo	Cu	Co	Fe	Na	S	Na	Cl
improved Hoagland’s medium (mM)	4	16	6.005	2	2	0.1	0.1	0.03	0.001	0.0001	0.0001	0.01	0.22375	2.2301	0.223885	0.0002
Hoagland’s solution without Ca^2+^ (mM)	0	16	11.005	3	2	0.1	0.1	0.03	0.001	0.0001	0.0001	0.01	0.22375	2.2301	0.223885	0.0002

The electrical conductivity (EC), which represent the concentration of the nutrient solution, was tested every 12 h and set to 1.6. The symptoms of tip-burn initially appeared at 3 d after treatment. The samples of leaves were harvested at 0, 3 and 6 d after treatment. The 0 d represent the plants not stressed with Ca^2+^ deficiency. For each treatment, 1 cm of leaf margin tissues were obtained from three individual plants and equally mixed as one sample. The leaf samples were immediately frozen with liquid nitrogen and then stored in an ultra-low temperature refrigerator at −80 °C. Each treatment was biologically replicated three times.

### Determination of physiological indexes and ABA concentration

The three treatment samples were used to test the physiological traits including superoxide dismutase (SOD, EC 1.15.1.1), peroxidase (POD, EC 1.11.1.7), catalase (CAT, EC 1.11.1.6) and ascorbate peroxidase (APX, EC 1.11.1.11), each treatment have three biologically replicated. The nitroblue tetrazolium reduced (NBT) method was used for SOD activity testing, guaiacol method was used for POD activity testing, hydrogen peroxide (H_2_O_2_) ultraviolet (UV) absorption method was used for CAT activity testing and ascorbic acid (AsA) oxidation rate method was used for APX activity testing. The 4 physiological indexes were tested using respective analytical reagent kit (Comin biotechnology Co., ltd. Suzhou, China) following the manufacturer’s procedure.

For ABA concentration tested, the samples were frozen in liquid nitrogen, ground into powder, extracted with methanol/water (8/2) at 4 °C and centrifuged at 12,000 g under 4 °C for 15 min. After that, collected supernatant fluid and evaporated to dryness under nitrogen gas stream. Then, samples were reconstituted in methanol/water (3/7), centrifuged and the supernatant was collected for high performance liquid chromatography (HPLC) according to its absorption peak at 254 nm^61^.

### Total RNA extraction and circRNA sequencing

The total RNAs were extracted from Chinese cabbage samples using TRIZOL reagent (Invitrogen Corp., Carlsbad, CA, USA) according to the manufacturer’s procedure. The total RNA purity, concentration and integrity were assessed on a NanoDrop ND-1000 spectrophotometer, Qubit 2.0 fluorometer quantitation platform and Agilent 2100 Bioanalyser Lab-on-Chip system, respectively.

The ribosomal RNAs were removed from total RNA according to the manuscript of the Epicentre Ribo-Zero Gold Kit (Illumina, San Diego, USA). The rRNA-depleted RNAs were further delete linear RNA using RNase R (Epicentre, Madison, WI) to. Then, the circRNA fragmentation was conducted using fragmentation buffer. The first-strand cDNA was synthesized using random hexamer primers, taking these fragment circRNAs as templates. Followed by the second-strand cDNA synthesized by adding dNTPs, DNA Polymerase I, RNase H and buffer. The T4 DNA and Klenow DNA polymerases were used to convert cohesive ends into blunt. After the adenylation of 3′ ends, the sequencing adaptors were ligated. The libraries were purified, and fragments sizes were chosen using AMPure XP Beads (Beckman Coulter, Beverly, USA). The USER enzyme (NEB, USA) was used to degrade the second-strand cDNA with U. Then, circRNA libraries were obtained by PCR amplification. The library effective concentration was detected using Qubit 2.0 and Q-PCR, and the library insert size was detected with an Agilent 2100. Then, the libraries were sequenced on the Illumina Hiseq-Xten platform by Biomarker Technologies with 150 bp paired-end reads.

### Identification and expression analysis circRNAs

To obtain clean reads, reads containing an adaptor, ploy-N and of low quality were removed from raw data. After that, the clean reads were mapped to the Chinese cabbage ‘Chifu-401-42’ genome sequence (http://brassicadb.org/brad/)^62^ with BWA software^63^. The circRNAs were accounted for using Find-circ software^64^ based on the follow standards: (1) both sides of splice site contain GU/AG; (2) breakpoints could be found; (3) only 2 mismatches are supported; (4) Breakpoint cannot occur outside anchor 2 nucleotides; (5) on junction must be mapped at least two reads; (6) the score of the blasting on the right position of short sequence is more than 35 points higher than that of other positions^65^.

The junction reads were used as gene expression levels. Differential expression analysis among the three treatments was performed using the edgeR package^66^. The circRNA was considered to be differentially expressed when fold change was >= 2 and P-value was < 0.05.

### CircRNA miRNA-binding sites analysis

In order to analyse miRNA sponge of circRNAs, we predicted miRNA–circRNA interactions of circRNAs. The TargetFinder software (https://github.com/carringtonlab/TargetFinder) was used to scan the miRNA-target sites of circRNA. The input files were circRNA FASTA sequences files and miRNA.

### CircRNA host genes functional annotation

The circRNA host genes’ functions were annotated based on the following databases: GO (Gene Ontology), KEGG (Kyoto Encyclopedia of Genes and Genomes), KOG/COG (Clusters of Orthologous Groups of Proteins), Pfam (Protein family), Swiss-Prot (A manually annotated and reviewed protein sequence database), eggNOG (Orthology predictions and functional annnotaion) and Nr (NCBI non-redundant protein sequences). The KEGG annotations were executed using blast software to align the host genes to KEGG databases. GO annotations were performed by BLAST2GO software with the default parameters. The enrichment analysis of GO and KEGG annotations of differentially expressed circRNAs’ host genes were implemented by Fisher exact test.

### Validation of expressed circRNAs

Nine circRNAs with high expression were selected to check the expression of the identified circRNAs in *B. rapa* using Quantitative real-Time PCR (qRT-PCR). First strand cDNA was synthesized from the RNA using TUREscript 1st Stand cDNA SYNTHESIS Kit (Aidlab Biotechnologies Co., Ltd). The primers were designed by Beacon Designer 7.9 software (Premier Biosoft International, Palo Alto, CA, USA) with junction sites contained in the amplifications, and the *B. rapa* 18 S rRNA was used as an internal control. The qRT-PCR was performed on a qTOWER 2.0/2.2 Quantitative Real-Time PCR Thermal Cyclers (Germany) using 2 × SYBR^®^ Green Master Mix (DBI) according to manufacturer’s manual. Transcript levels were calculated using the 2^−ΔΔCt^ method^67^. Three biological replicates were performed on each sample and three technical replicates were included on each biological replicate. We also analyzed the correlation between the expression level of circRNAs detected by CircRNA-Seq and qRT-PCR.

### Data analysis

Analysis of variance post hoc multiple comparison test (LSD’s honestly significant difference, P < 0.05) were performed using the statistical software SPSS version 16.0 software. The pictures of enzyme activities were drawn in Microsoft Excel 2016.

## Supplementary information

Supplementary Table S1

Supplementary Table S2

Supplementary Table S3

Supplementary Table S4

Supplementary Table S5

Supplementary Table S6

Supplementary Table S7

Supplementary Table S8
